# Supplementary material for: Trapped-ion quantum simulation of electron transfer models with tunable dissipation
Source: Sci Adv. 2024 Dec 20;10(51):eads8011. doi: 10.1126/sciadv.ads8011 (PMC11661436; doi:10.1126/sciadv.ads8011)
Supplement: Supplementary file 1 — Supplementary sections S1 to S6 Figs. S1 to S4 References [file sciadv.ads8011_sm.pdf]

Science Advances
AAAS

Supplementary Materials for

Trapped-ion quantum simulation of electron transfer models with tunable dissipation

Visal So *et al.*

Corresponding author: Guido Pagano, pagano@rice.edu

Sci. Adv. **10**, eads8011 (2024)
DOI: 10.1126/sciadv.ads8011

This PDF file includes:

Supplementary sections S1 to S6
Figs. S1 to S4
References

S1 Hamiltonian derivation

In this section, we derive the mapping from the experimental trapped-ion Hamiltonian to the electron transfer model in Eq. (1) in the main text. When we apply a pair of counter-propagating Raman beams with a wavevector difference of $\vec{k}$, phase difference of ϕ, and a beatnote frequency at ω_L on the $^{171}Yb^+$ trapped qubit in a dual-species chain, the system can be described by ($\hbar = 1$)

$$H = \frac{\omega_{\text{hf}}}{2}\sigma_z + \sum_\nu \omega_\nu a_\nu^\dagger a_\nu + \frac{\Omega}{2}\left(e^{\sum_\nu i\eta_\nu\left(a_\nu + a_\nu^\dagger\right) - i\omega_L t - i\phi}\sigma^+ + \text{h.c.}\right), \tag{S.1}$$

where ω_{hf} is the energy splitting between the two qubit states, ω_ν is the ν-th collective motional frequency of the chain associated with the raising (lowering) operator, $a_\nu^\dagger(a_\nu)$, Ω is the Rabi coupling strength, and $\eta_\nu = k\sqrt{1/2m\omega_\nu} b_\nu$ is the Lamb-Dicke parameter, and m is the qubit mass. b_ν is the normalized motional eigenvector for the $^{171}Yb^+$ qubit ion in the $\nu = 1, 2$ modes, namely the com and tilt modes of the $^{171}Yb^+$ -$^{172}Yb^+$ crystal.

By adding and subtracting $\sum_\nu \delta_\nu a_\nu^\dagger a_\nu$ to Eq. (S.1) and rotating with respect to $\frac{\omega_{\text{hf}}}{2}\sigma_z + \sum_\nu \mu a_\nu^\dagger a_\nu$, Eq. (S.1) is transformed into a resonant interaction frame rotating at $\mu = \omega_L - \omega_{\text{hf}} \equiv \omega_\nu + \delta_\nu$, where δ_ν is the detuning from the ν-th motional mode *(41)*. In our experiment, $\mu + \omega_\nu \gg |\mu - \omega_\nu| = \delta_\nu$, therefore a rotating-wave approximation (RWA) is justified, and terms that rotate at $\mu + \omega_\nu$ can be neglected. After the RWA, the Hamiltonian is described by

$$H_I^{\text{res}} = \frac{\Omega}{2}\left(e^{i\sum_\nu \eta_\nu\left(a_\nu e^{-i\mu t} + a_\nu^\dagger e^{i\mu t}\right)} e^{-i(\omega_L - \omega_{\text{hf}})t - i\phi}\sigma^+ + \text{h.c.}\right) - \sum_\nu \delta_\nu a_\nu^\dagger a_\nu. \tag{S.2}$$

For our setup, the detuning from the tilt mode ($\delta_{\text{tilt}}/2\pi \equiv \delta/2\pi$ in the main text) ranges from -4 to -10 kHz, while $\delta_{\text{com}}/2\pi \sim -160\,\text{kHz}$. Therefore, we can safely neglect the contribution from the com mode, obtaining a single-mode Hamiltonian:

$$H_I^{\text{res}} = \frac{\Omega}{2}\left(e^{i\eta\left(a e^{-i\mu t} + a^\dagger e^{i\mu t}\right)} e^{-i(\omega_L - \omega_{\text{hf}})t - i\phi}\sigma^+ + \text{h.c.}\right) - \delta a^\dagger a. \tag{S.3}$$

In the experiment, we apply 4 tones to one of the Raman beams generating four beatnotes at

frequencies $\omega_r = \omega_{\text{hf}} - \mu$ with phase ϕ_r (red sideband or RSB), $\omega_b = \omega_{\text{hf}} + \mu$ with phase ϕ_b (blue sideband or BSB), $\omega_x = \omega_{\text{hf}}$ with phase ϕ_x, and $\omega_y = \omega_{\text{hf}}$ with phase ϕ_y. Thus, Eq. (S.3) becomes

$$H_I^{\text{res}} = \sum_{k=r,b,x,y} \frac{\Omega_k}{2} \left(e^{i\eta\left(ae^{-i\mu t} + a^\dagger e^{i\mu t}\right)} e^{-i(\omega_k - \omega_{\text{hf}})t - i\phi_k} \sigma^+ + \text{h.c.} \right) - \delta a^\dagger a, \tag{S.4}$$

The first two terms in the summation generate the spin-phonon coupling term in Eq. (1) in the main text. In the Lamb-Dicke regime, where $\eta\sqrt{\langle (a + a^\dagger)^2 \rangle} \ll 1$, we can expand the two terms with respect to η to the first order and apply rotating-wave approximation to neglect off-resonant terms rotating at μ and 2μ. For $\Omega_r = \Omega_b \equiv \Omega$, we obtain the effective spin-phonon Hamiltonian:

$$H_{\text{sp}} = \frac{\eta\Omega}{2} \left(ae^{i\phi_m} + a^\dagger e^{-i\phi_m} \right) \left(\cos\phi_s \sigma_x + \sin\phi_s \sigma_y \right), \tag{S.5}$$

where the motional phase $\phi_m \equiv \frac{\phi_b - \phi_r}{2}$ and the spin phase $\phi_s \equiv \frac{\phi_b + \phi_r}{2} - \frac{\pi}{2}$ *(57)*. We choose $\phi_r = \phi_b = \pi$ for the experiment. Hence, the Hamiltonian is further simplified to

$$H_{\text{sp}} = \frac{\eta\Omega}{2} \sigma_y \left(a + a^\dagger \right). \tag{S.6}$$

The two remaining terms in the summation in Eq. (S.4) follow the same form, differed by only the phase difference ϕ_k with $k = x, y$ and, in the Lamb-Dicke regime, generate

$$H_k = \frac{\Omega_k}{2} \left(\cos\phi_k \sigma_x + \sin\phi_k \sigma_y \right). \tag{S.7}$$

By substituting Eq. (S.6) and Eq. (S.7) with $\phi_x = 0$ and $\phi_y = \pi/2$ into Eq. (S.4), we obtain

$$H_I^{\text{res}} = \frac{\Omega_y}{2} \sigma_y + \frac{\Omega_x}{2} \sigma_x + \frac{\eta\Omega}{2} \sigma_y (a^\dagger + a) - \delta a^\dagger a \tag{S.8}$$

As explained in the main text, to map Eq. (S.8) to the ET model in Eq. (1) in the main text, we apply a rotation $U_x(\pi/2) = \exp(-i\sigma_x \pi/4)$ to the qubit initialized in $|{\downarrow}\rangle_z$ prior to the simulation (see Fig. 2 in the main text). This rotates the qubit to $|{\uparrow}\rangle_y$. At the end of

the evolution, we apply another rotation $U_x(\pi/2)$ to perform the mapping $|\uparrow\rangle_y \leftrightarrow |\uparrow\rangle_z$ and $|\downarrow\rangle_y \leftrightarrow |\downarrow\rangle_z$. Therefore, to realize the electron transfer Hamiltonian in Eq. (1) in the main text, the parameter mappings are $\Omega_y = \Delta E$, $\Omega_x/2 = V_x$, $\eta\Omega = g$, and $\delta = -\omega$.

S2 System calibration

We independently calibrate the parameters of the laser tones used to realize both the unitary and the dissipative terms in Eq. (2) in the main text. The spin-phonon coupling and phonon terms, $\frac{\eta\Omega}{2}\sigma_y(a^\dagger + a) - \delta a^\dagger a$, are calibrated by adjusting the Rabi frequency $\eta\Omega_n$ and detunings δ_n of the red and blue sideband Raman laser beatnotes from the tilt mode sideband resonances with $n = r, b$.

We calibrate the spin-phonon coupling and the detuning by preparing the ^{171}Yb$^+$ qubit in the z basis and applying the Hamiltonian in Eq. (7) in the main text with $\eta\Omega$ as the spin-phonon Rabi coupling strength. The hopping period corresponds to $2\pi/|\delta|$, which we use to estimate δ. We then drive each tone on resonance to the tilt mode separately while setting the other tone off-resonant to estimate the effective $\eta\Omega$ for the experiments (see Figs. S1A-B). In order to compensate for the ac-Stark shift due to the off-resonant excitation of the carrier transition by the red and blue sidebands, we use the following procedure: we first balance the Rabi coupling strengths of the red and blue sideband resonant drives ($\delta_n = 0$) to the tilt mode separately. Then we turn on both tones simultaneously with the same detuning, $\delta_r = \delta_b = \delta$, from the tilt mode resonances. Using a Ramsey sequence, we adjust the ratio of the powers and a common frequency shift of the two laser tones to compensate for the undesired ac-Stark shift up to 0.25 kHz accuracy. For the Ω_x and Ω_y Rabi frequencies in Eq. (S.8), we adjust the power of the two carrier transition tones independently (see Fig. S1C).

The frequency of the 435 nm red sideband resonance of the ^{172}Yb$^+$ optical transition is found by using a scheme similar to Quantum logic spectroscopy (QLS) via spin-state measure-

ments of $^{171}\mathrm{Yb}^+$. The pulse sequence consists of a series of three π-pulses on both ions, namely $\mathrm{BSB}\pi(355\mathrm{nm}) \rightarrow \mathrm{RSB}\pi(435\mathrm{nm}) \rightarrow \mathrm{BSB}\pi$(355 nm), while varying the frequency of the 435 nm RSBπ pulse. The 355 nm light is kept on and out of resonance with the $^{171}\mathrm{Yb}^+$ qubit during 435 nm illumination to account for the differential stark shift on the 435 nm cooling transition. Another method to quantify the 435 nm cooling transition frequency is to replace the initial sideband cooling (SBC) pulses of the tilt mode with a finite amount of 435 nm continuous sideband cooling (CSBC) pulse. We then scan the RSB frequency of both the Zeeman $\Delta m_j = 0$ transitions of $^{172}\mathrm{Yb}^+$ parking at 2π time of the tilt mode BSB evolution. By observing the contrast of the BSB population, we estimate the stark shifted frequencies of the 435 nm RSB pulse used for sympathetic cooling.

To estimate the cooling rate γ and the steady-state phonon $\bar{n}$ with 435 nm and 935 nm beams on $^{172}\mathrm{Yb}^+$, we carry out the following procedure: we first perform Doppler cooling on the dual-species chain; then, we employ continuous sideband cooling on the tilt mode through $^{172}\mathrm{Yb}^+$, followed by Raman sideband cooling on the com mode through $^{171}\mathrm{Yb}^+$; subsequently, we optically pump the spin state of $^{171}\mathrm{Yb}^+$ to $|\downarrow\rangle_z$ and perform a phonon distribution measurement on tilt mode via a resonant BSB drive to estimate the average tilt mode phonon. By varying the cooling time and measuring the corresponding average phonon, we can obtain the cooling rate γ and the average phonon $\bar{n}$ in Eq. (2) in the main text with an exponential fit, as shown in Fig. S1D. The cooling rate can be adjusted by changing the 935 nm repumper power as it is non-monotonically dependent on the 935 nm power exhibiting an optimum, as shown in Fig. S2.

S3 Numerical simulations

We simulate Eq. (2) in the main text using a Python package based on QUTIP *(63)*, which allows us to include experimental imperfections that induce different types of dephasing in our

system. As the experiment is performed in the rotated basis ($z \leftrightarrow y$), fluctuations in the laser intensity and detuning cause effective spin decoherence, while trap frequency fluctuations and the heating rate of tilt motional mode cause motional dephasing. Therefore, when comparing the numerics with the experimental results, we introduce two additional dissipative processes, which modify Eq. (2) in the main text to

$$\frac{\partial \rho}{\partial t} = -i[H_{\rm s}, \rho] + \gamma(\bar{n}+1)\mathcal{L}_a[\rho] + \gamma\bar{n}\mathcal{L}_{a^\dagger}[\rho] + \sum_{k=z,m} \gamma_k \mathcal{L}_{c_k}[\rho], \tag{S.9}$$

where the jump operator $c_z = \sigma_y$ and its corresponding rate γ_z account for spin decoherence while the jump operator $c_m = aa^\dagger + a^\dagger a$ and its corresponding rate γ_m account for motional dephasing *(64)*. We determine these dephasing rates by comparing numerical results to experimental data, finding that $\gamma_z/2\pi \sim (0-10)$ Hz, and $\gamma_m/2\pi \sim 5$ Hz.

S4 Electron transfer transient dynamics

As discussed in the main text, the spin evolutions in the non-adiabatic and strongly adiabatic regimes exhibit different behaviors. In Fig. S3, we report the time traces of the donor population evolution for $\Delta E = \omega/2, \omega, 2\omega$ in both regimes. As shown in Figs. S3A-B, the dynamics of the donor population in the non-adiabatic regime can be modeled with an exponential decay. On the other hand, the dynamics in the strongly adiabatic regime feature complex oscillatory decays that cannot be fitted with an exponential decay as those in the non-adiabatic regime (see Figs. S3C-D). In the strongly adiabatic regime, when $\Delta E = \omega$, the oscillations are evident because the effective vibronic coupling is dominated by the $V_x\sqrt{\mathrm{FC}_{0_-,1_+}}$ coupling term, which has the largest Franck-Condon factor. Meanwhile, in the $\Delta E = \omega/2$ case, the oscillations are less pronounced because of the non-integer value of $\Delta E/\omega$ that suppresses the transfer, analogous to off-resonant coupling in a two-level system (see Fig. S3C). Finally, when $\Delta E = 2\omega$, the relevant Franck-Condon factor $\mathrm{FC}_{0_-,2_+}$ is smaller than the $\Delta E = \omega$ case, which decreases

the effective vibronic coupling, resulting in both smaller frequency and amplitude of the oscillations.

S5 Phonon steady state characterization

In addition to the spin degrees of freedom, a trapped-ion platform has the inherent capability of measuring motional observables, allowing access to spin-phonon correlations as well as to models in which the motional degrees of freedom govern the dynamics. For example, the observations of the destructive interference of the wavepacket due to the geometric phase in the quantum simulation of conical intersections are performed through the reconstruction of the ion's wavefunction spatial distribution *(21, 22)*.

Although the motional degree of freedom only plays a surrogate role in the dynamics of the electron transfer model studied here, we note that, from a quantum optics perspective, our system simulates a variant of the Rabi model *(35)* with tunable dissipation, ranging from weak to ultra-strong coupling regimes. In this regard, the Rabi model with dissipation can be investigated by measuring both motional and spin observables. In this work, we study the electron transfer models in the $g \gg \gamma$ regime, where the dynamics of the spin degree of freedom is inherently non-Markovian. Therefore, the spin and the oscillator can have finite correlations in the steady state *(65)*. Specifically, in the non-adiabatic regime at $\Delta E = 0$, the steady state of the ET system is a classical ensemble of donor and acceptor vibronic states with equal weights. Meanwhile, the steady state in the strongly adiabatic regime at $\Delta E = 0$ is a quantum superposition of donor and acceptor vibronic states with equal probabilities. As ΔE increases, the ensemble weight or quantum probability of the acceptor vibronic state increases towards 1 in both cases. Such a change in the steady-state structure can be captured by a decrease in the spin-phonon correlation, which in turn affects also the steady-state phonon population. Here, we measure the average phonon population in the steady state of the evolution under Eq. (2) in

the main text and indirectly observe spin-phonon correlations depending on the donor-acceptor energy separation ΔE.

First, we discuss the steady state of the Lindbladian master equation, focusing in particular on the properties of the phonon population. From Eq. (2) in the main text, the expectation of an observable O satisfies

$$\begin{aligned}\partial_t\langle O\rangle =& \mathrm{Tr}\left[-iO\left[H,\rho\right]+O\gamma\left(\bar{n}+1\right)\mathcal{D}_a\left(\rho\right)+O\gamma\bar{n}\mathcal{D}_{a^\dagger}\left(\rho\right)\right]\\ =& -i\langle[O,H]\rangle+\gamma\left(\bar{n}+1\right)\langle a^\dagger O a-\frac{1}{2}\left\{O,a^\dagger a\right\}\rangle\\ &+\gamma\bar{n}\langle aOa^\dagger-\frac{1}{2}\left\{O,aa^\dagger\right\}\rangle.\end{aligned} \tag{S.10}$$

Using the bosonic commutation relation, the number operator $n=a^\dagger a$ satisfies

$$\partial_t\langle n\rangle=-i\frac{g}{2}\langle\sigma_z\left(a^\dagger-a\right)\rangle+\gamma\left(\bar{n}-\langle n\rangle\right). \tag{S.11}$$

The creation/annihilation operators satisfy

$$\begin{aligned}\partial_t\langle a^\dagger\rangle &= i\frac{g}{2}\langle\sigma_z\rangle+\left(i\omega-\gamma/2\right)\langle a^\dagger\rangle,\\ \partial_t\langle a\rangle &= -i\frac{g}{2}\langle\sigma_z\rangle-\left(i\omega+\gamma/2\right)\langle a\rangle.\end{aligned} \tag{S.12}$$

To obtain steady-state solutions, we set LHS of Eq. (S.11), (S.12) equal to zero leading to

$$n_{\mathrm{ss}}=\bar{n}-\frac{i}{2}\frac{g}{\gamma}\langle\sigma_z\left(a^\dagger-a\right)\rangle_{\mathrm{ss}}, \tag{S.13}$$

$$\langle a^\dagger\rangle_{\mathrm{ss}}=-\frac{ig}{2i\omega-\gamma}\langle\sigma_z\rangle_{\mathrm{ss}},\quad \langle a\rangle_{\mathrm{ss}}=-\frac{ig}{2i\omega+\gamma}\langle\sigma_z\rangle_{\mathrm{ss}}, \tag{S.14}$$

which immediately gives an expression of steady-state reaction coordinate y_{ss} in terms of the steady-state donor population P_D^{ss} as follows

$$y_{\mathrm{ss}}=\frac{y_0}{2}\left(a+a^\dagger\right)=-\frac{2\omega g}{4\omega^2+\gamma^2}(2P_D^{\mathrm{ss}}-1)y_0. \tag{S.15}$$

To quantify spin-phonon correlation, we can compare the exact steady-state $n_{\rm ss}$ in Eq. (S.13) with the one calculated assuming that spin and phonon are in an uncorrelated state, namely $\langle\sigma_z\left(a^\dagger - a\right)\rangle_{\rm ss} = \langle\sigma_z\rangle_{\rm ss}\langle a^\dagger - a\rangle_{\rm ss}$, which leads to

$$n_{\rm ss}^{\rm un} = \bar{n} + \frac{g^2}{4\omega + \gamma^2}\left(2P_D^{\rm ss} - 1\right)^2. \tag{S.16}$$

In Fig. S4, we report measurements of the steady-state $\langle a^\dagger a\rangle_{\rm ss}$ as a function of ΔE in the case of ultra-strong coupling ($g/\omega = 1.91$). To measure the steady-state phonon population, the qubit is reset via optical pumping, followed by the application of a resonant BSB Hamiltonian $H^{\rm BSB} = i(\eta\Omega/2)(a\sigma^- - a^\dagger\sigma^+)$ to the system after the evolution has reached its steady state. The resulting spin evolution is then fitted to extract the average phonon population $\langle n\rangle_{\rm ss} = \langle a^\dagger a\rangle_{\rm ss}$ in the steady state. The measured values are in agreement with the exact solution $\langle n\rangle_{\rm ss}$ in Eq. (S.13), confirming the presence of spin-phonon correlations in the system's steady state.

Furthermore, to get an intuitive understanding of the effect of the dissipation due to the Lindbladian, we shall assume the spin is either in $|\uparrow\rangle$ or $|\downarrow\rangle$ such that the Hamiltonian in Eq. (1) in the main text can be reduced to

$$H_p = \omega a^\dagger a \pm \frac{g}{2}(a + a^\dagger). \tag{S.17}$$

The corresponding master equation becomes

$$\partial_t\rho = -i[H_p, \rho] + \gamma(\bar{n}+1)\mathcal{D}_a[\rho] + \gamma\bar{n}\mathcal{D}_{a^\dagger}[\rho]. \tag{S.18}$$

We can define displaced bosonic creation/annihilation operators $b \equiv a + \alpha, b^\dagger \equiv a^\dagger + \alpha^*$ with α being a complex constant to be determined. It can be shown that if we set

$$\alpha = \pm\frac{2g\omega}{4\omega^2 + \gamma^2} \pm \frac{ig\gamma}{4\omega^2 + \gamma^2}, \tag{S.19}$$

Eq. (S.18) then becomes

$$\partial_t\rho = -i[\omega b^\dagger b, \rho] + \gamma(\bar{n}+1)\mathcal{D}_b[\rho] + \gamma\bar{n}\mathcal{D}_{b^\dagger}[\rho], \tag{S.20}$$

which takes the form of a simple damped oscillator with steady state being a thermal vibrational state characterized by $\bar{n}$ *(66)*. Undoing the displacement gives us the steady-state solution of Eq.(S.18):

$$\rho_{ss} = D(-\alpha)\rho_{th}D(\alpha),\ \rho_{th} = \frac{e^{-\beta\omega a^{\dagger}a}}{1 - e^{-\beta\omega}}, \tag{S.21}$$

where D is the displacement operator and $1/\beta = \omega/\log(1 + 1/\bar{n})$. In the experiment, $\omega \gg \gamma$ such that $\alpha \to \pm g/2\omega$. Depending on the spin, the system is effectively pumped to the ground state of the left/right displaced harmonic well, as shown in Fig. 1B.

S6 Ohmic bath and Lindbladian formalism

In this section, we show that the derivation of a Lindbladian master equation for the system in Eq. (1) in the main text in contact with an Ohmic bath is equivalent to the dissipative spin-boson model realized in this experiment under certain conditions. More formally, we will establish the equivalence between Eq. (2) in the main text and the spin-boson Hamiltonian H_{ET} in Eq. (1.3) of *(31)*, namely

$$H_{ET} = H_{\text{s}} + H_{\text{sb}} + H_{\text{b}}. \tag{S.22}$$

The system Hamiltonian H_{s} is given by Eq. (1) in the main text. The bath Hamiltonian $H_{\text{b}} = \sum_n \omega_n \Gamma_n^{\dagger}\Gamma_n$ is described by a collection of infinite harmonic oscillators with $\Gamma_n(\Gamma_n^{\dagger})$ being the annihilation(creation) operator of the n-th bosonic mode. The reaction coordinate of the system is linearly coupled to the position coordinate of the bath via

$$H_{\text{sb}} = S \otimes B,\ S \equiv a + a^{\dagger},\ \ B \equiv K + K^{\dagger}, \tag{S.23}$$

with $K^{\dagger} \equiv \sum_n c_n \Gamma_n^{\dagger}$ being a linear combination of bath operators and c_n being the coupling coefficients of the n-th mode. The coupling coefficients c_n and the bath frequencies determine the bath spectral density function $J(\omega) = \sum_n c_n^2 \delta(\omega - \omega_n)$. We take $J(\omega)$ to be Ohmic *(31)*:

$$J(\omega) = \eta\omega \exp(-\omega/\omega_c), \omega_c \to \infty, \tag{S.24}$$

which corresponds to a classical damped oscillator with η being the linear damping coefficient. Note that in this section, we use ω as the frequency variable for the spectral density function J and ω_0 as the bosonic mode frequency in Eq. (1) in the main text.

To obtain the reduced dynamics of the system density matrix $\rho(t)$, we shall first change into the interaction picture of $H_0 = H_{\rm s} + H_{\rm b}$. Let us denote a generic operator O in the interaction picture of H_0 as

$$\widetilde{O}\,(t) = U^{\dagger}\,(t)\,OU\,(t)\,,\;\; U(t) = \exp\left(-iH_0 t\right), \tag{S.25}$$

the master equation in the interaction picture is then given by

$$\partial_t \widetilde{\chi}\,(t) = -i[\widetilde{H}_{\rm sb}(t), \widetilde{\chi}\,(t)], \tag{S.26}$$

where χ is the system-bath density matrix. Explicitly integrating this equation and inserting the expression for $\widetilde{\chi}$ back leads to

$$\partial_t \widetilde{\chi}\,(t) = -i[\widetilde{H}_{\rm sb}\,(t)\,, \chi\,(0)] - \int_0^t dt' [\widetilde{H}_{\rm sb}(t), [\widetilde{H}_{\rm sb}(t'), \chi\,(t')]]. \tag{S.27}$$

Assuming the system-bath coupling is sufficiently weak, and the bath is kept at thermal equilibrium, $\chi(t)$ becomes separable (Born approximation):

$$\chi\,(t) = \rho(t) \otimes R_0, \quad R_0 = e^{-\beta H_{\rm b}}/\mathrm{Tr}(e^{-\beta H_{\rm b}}), \tag{S.28}$$

where $\beta = 1/k_B T$. Taking partial trace with respect to the bath degrees of freedom on Eq. (S.27) gives an equation for $\widetilde{\rho}$:

$$\begin{aligned}\partial_t \widetilde{\rho}\,(t) = -\int_0^t & dt' C\,(\tau) \left[\widetilde{S}\,(t)\,\widetilde{S}\,(t')\,\widetilde{\rho}\,(t') - \widetilde{S}\,(t')\,\widetilde{\rho}\,(t')\,\widetilde{S}\,(t)\right] \\ & + C\,(-\tau) \left[\widetilde{\rho}\,(t')\,\widetilde{S}\,(t')\,\widetilde{S}\,(t) - \widetilde{S}\,(t)\,\widetilde{\rho}\,(t')\,\widetilde{S}\,(t')\right],\end{aligned} \tag{S.29}$$

where $\tau \equiv t - t'$ and $C(\tau)$ is the temporal correlation function of the bath, namely

$$\begin{aligned} C\,(\tau) &= \mathrm{Tr}\left[\widetilde{B}\,(\tau)\,BR_0\right] \\ &= \int_0^\infty d\omega J\,(\omega) \left[\coth\left(\beta\omega/2\right)\cos\left(\omega\tau\right) - i\sin\left(\omega\tau\right)\right]. \end{aligned} \tag{S.30}$$

$S, \widetilde{S}, B, \widetilde{B}$ follow the definitions in Eq. (S.23),(S.25).

By introducing a displaced bosonic operator $b = a + a_0$ with the spin dependent constant $a_0 = \frac{g}{2\omega_0}\sigma_z$, we can evaluate the interaction frame system operator $\widetilde{S}(t)$:

$$\widetilde{S}\left(t\right) = \left(a^{\dagger} e^{i\omega_0 t} + a e^{-i\omega_0 t}\right) + 2a_0 \left(\cos\omega_0 t - 1\right). \tag{S.31}$$

Assuming the bath correlation function is strongly peaked around $\tau = 0$ with a correlation time τ_r much smaller than the typical time scale of the system's dynamics, $C(\tau)$ can be approximated as $\delta(\tau)$, which yields the replacement $\rho\left(t'\right) \rightarrow \rho\left(t\right)$. Another important effect of this approximation is the extension of the integration limit from t to ∞ of the integral in Eq. (S.29) (Markovian approximation) *(67)*. Eq. (S.29) then results in the Schrodinger picture master equation:

$$\begin{aligned}
\partial_t \rho\left(t\right) = &- i\left[H_{\mathrm{s}}, \rho\left(t\right)\right] - \int_0^{\infty} d\tau C\left(\tau\right)\left[S\widetilde{S}\left(-\tau\right)\rho\left(t\right) - \widetilde{S}\left(-\tau\right)\rho\left(t\right)S\right] \\
&+ \int_0^{\infty} d\tau C\left(-\tau\right)\left[\rho\left(t\right)\widetilde{S}\left(-\tau\right)S - S\rho\left(t\right)\widetilde{S}\left(-\tau\right)\right].
\end{aligned} \tag{S.32}$$

Inserting Eq. (S.30) and Eq. (S.31), this equation can be written in a compact form as

$$\partial_t \rho\left(t\right) = -i\left[H_{\mathrm{s}}, \rho\left(t\right)\right] - \left[S(\Lambda + C_0)\rho\left(t\right) - (\Lambda + C_0)\rho\left(t\right)S + \mathrm{h.c.}\right], \tag{S.33}$$

where $\Lambda = \mathcal{L}_+ a + \mathcal{L} a^{\dagger}$ and $\mathcal{L}_+, \mathcal{L}$ take form of Laplace transform:

$$\begin{aligned}
\mathcal{L}_+ &= \int_0^{\infty} d\omega J\left(\omega\right)\left[1 + \bar{n}\left(\omega\right)\right] \int_0^{\infty} d\tau e^{-i(\omega-\omega_0)\tau}, \\
\mathcal{L} &= \int_0^{\infty} d\omega J\left(\omega\right)\bar{n}\left(\omega\right) \int_0^{\infty} d\tau e^{i(\omega-\omega_0)\tau},
\end{aligned} \tag{S.34}$$

where $\bar{n}(\omega)$ is the thermal phonon population of a mode of frequency ω and C_0 is a constant due to the scalar part of Eq. (S.31),

$$C_0 = 2a_0 \int_0^{\infty} d\tau C\left(\tau\right)\left(\cos\omega_0\tau - 1\right). \tag{S.35}$$

It shall be noted that in the evaluation of Λ, we have applied the secular approximation, neglecting the contribution from highly oscillatory terms involving $e^{\pm i(\omega+\omega_0)t}$ *(68)*.

Evaluating the integrals in Eq. (S.34), (S.35) leads to the reduced master equation of the system:

$$\begin{aligned}\partial_t\rho =& -i\left[H_{\mathrm{s}}+H_c,\rho\right]-i\left[H_n\rho-\rho H_n^\dagger\right]\\&+\gamma\left(\bar{n}_0+1\right)\left(\mathcal{D}_a\left(\rho\right)+\mathcal{D}'_a\left(\rho\right)\right)\\&+\gamma\bar{n}_0\left(\mathcal{D}_{a^\dagger}\left(\rho\right)+\mathcal{D}'_{a^\dagger}\left(\rho\right)\right)\\&+i\Delta_d\mathcal{D}_a^{Im}\left(\rho\right).\end{aligned}\tag{S.36}$$

Let us break down the different terms of the master equation (S.36): $\mathcal{D}_c(\rho)$ is the Lindbladian super-operator defined in Eq. (3) in the main text with coefficients,

$$\gamma=2\pi\eta\omega_0,\ \ \bar{n}_0=\bar{n}(\omega_0).\tag{S.37}$$

$\mathcal{D}'_c(\rho)$ is defined as

$$\mathcal{D}'_c(\rho)\equiv\frac{1}{2}\left(c^\dagger\rho c^\dagger+c\rho c-c^\dagger c^\dagger\rho-\rho cc\right),\tag{S.38}$$

$\mathcal{D}_a^{Im}\left(\rho\right)$ represents a super-operator with imaginary coefficients

$$\mathcal{D}_c^{Im}\left(\rho\right)\equiv\left(c\rho c-c^\dagger\rho c^\dagger\right),\tag{S.39}$$

H_n represents the following non-hermitian Hamiltonian:

$$H_n=\Delta_d aa,\ \Delta_d=P\left[\int_0^\infty d\omega\frac{(2\bar{n}\left(\omega\right)+1)J\left(\omega\right)}{\omega_0-\omega}\right],\tag{S.40}$$

where P stands for Principal Value, and H_c is a correction to the system Hamiltonian:

$$\begin{aligned}H_c&=\widetilde{\omega}a^\dagger a+\frac{\widetilde{g}}{2}\sigma_z\left(a+a^\dagger\right),\\\widetilde{\omega}&=P\left[\int_0^\infty d\omega\frac{J\left(\omega\right)}{\omega_0-\omega}\right],\\\widetilde{g}&=4gP\left[\int_0^\infty d\omega\frac{J\left(\omega\right)}{\omega_0^2-\omega^2}\right].\end{aligned}\tag{S.41}$$

When the frequency of the system's oscillator ω_0 is much larger than the decay rate γ, under rotating wave approximation, we can effectively neglect the terms involving $aa, a^\dagger a^\dagger$ that do not conserve the energy *(67)*. These include the super-operators $\mathcal{D}'_a(\rho), \mathcal{D}'_{a^\dagger}(\rho), \mathcal{D}^{Im}_a(\rho)$ and the non-hermitian Hamiltonian terms H_n. The rotating wave approximation is consistent with the Born approximation, which assumes that the system-bath coupling is sufficiently small so that the system and bath can be described by a separable state. After the above approximations, (S.36) takes form of a standard Lindbladian master equation *(69, 70)*, and we obtain Eq. (2) in the main text, with renormalized oscillator frequency $\omega' = \omega_0 + \widetilde{\omega}$ and displacement $g' = g + \widetilde{g}$.

We can check the validity of the Markovian approximation by comparing the time scale of the system dynamics $\tau_\mathrm{s} \sim 1/\gamma$ and the width of the position correlation function estimated by the bath correlation time $\tau_r \sim \beta$ *(67)*. For the approximation to be valid, we therefore require $\tau_r \ll \tau_\mathrm{s}$. Hence, for Eq. (2) in the main text to be a good description of the ET model in the weak decay regime, we require the following conditions, which are also derived in *(40)*:

$$\gamma \ll 1/\beta, \qquad \text{(Markovian)}$$

$$\gamma \ll \omega_0. \qquad \text{(RWA, Born)}$$

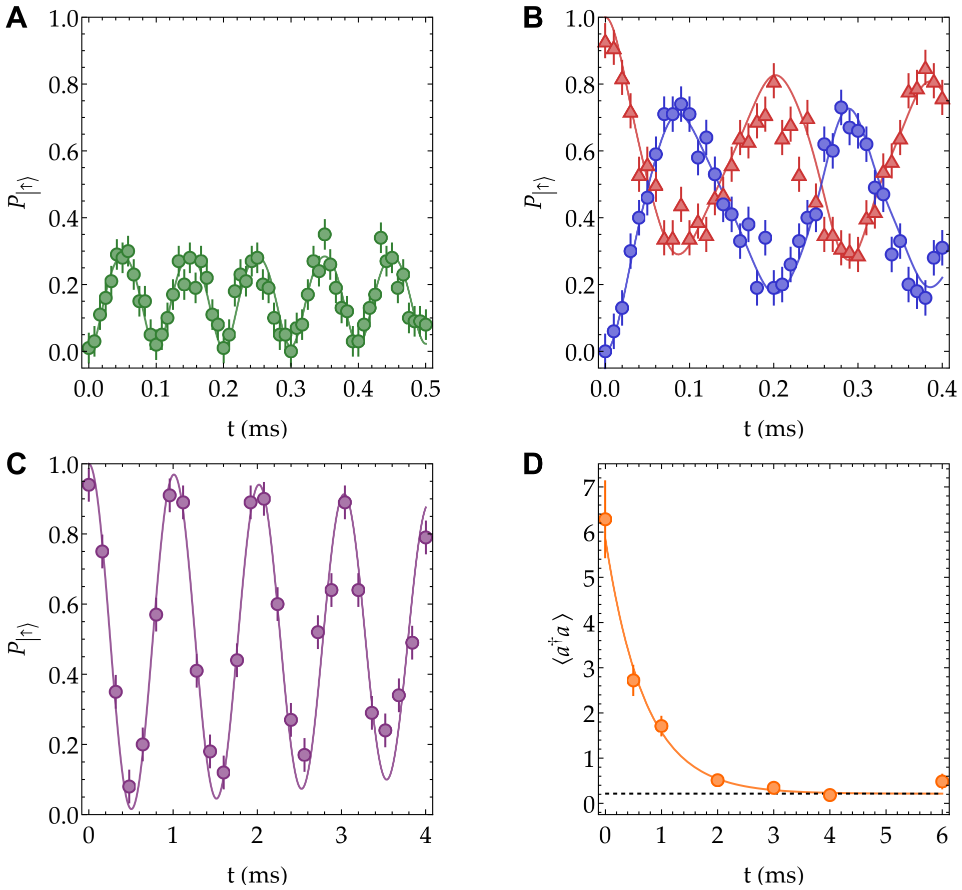

Figure S1: **Hamiltonian and dissipation experimental calibration.** (**A**) Spin dynamics from the red and blue sideband Raman laser beatnotes with a common detuning from the tilt mode, $\delta/2\pi = -10$ kHz, and equal Rabi coupling strengths, $\eta\Omega = 0.55|\delta|$. The hopping period corresponds to $2\pi/|\delta|$. (**B**) The same spin-phonon drives with one tone on resonance and another detuned from the tilt mode. Red triangles (experimental data) and solid curve (numerics) correspond to $\{\delta_r/2\pi, \delta_b/2\pi\} = \{0, -10\}$ kHz, and the blue circles correspond to $\{\delta_r/2\pi, \delta_b/2\pi\} = \{-10, 0\}$ kHz. (**C**) Spin dynamics undergoes a carrier drive along x in the σ_y basis. The Rabi coupling strength is set to $\Omega_x/2\pi = 0.99$ kHz. Together with another tone of the same frequency beatnote and phase difference of $\pi/2$, we generate the spin operation terms in the electron transfer Hamiltonian. The numerical results represented by solid curves in (A)-(C) include spin decoherence ($\gamma_z/2\pi = 10$ Hz) and motional dephasing ($\gamma_m/2\pi = 5$ Hz). (**D**) The evolution of the average tilt mode phonon occupation number of the dual-species ion crystal via continuous resolved sideband cooling on $^{172}Yb^{+}$ with 435 nm and 935 nm beams. The exponential constant determines the cooling rate. Here, $\gamma/2\pi = 0.23$ kHz, and the steady state average phonon occupation number is $\bar{n} = 0.2$ (dashed horizontal line).

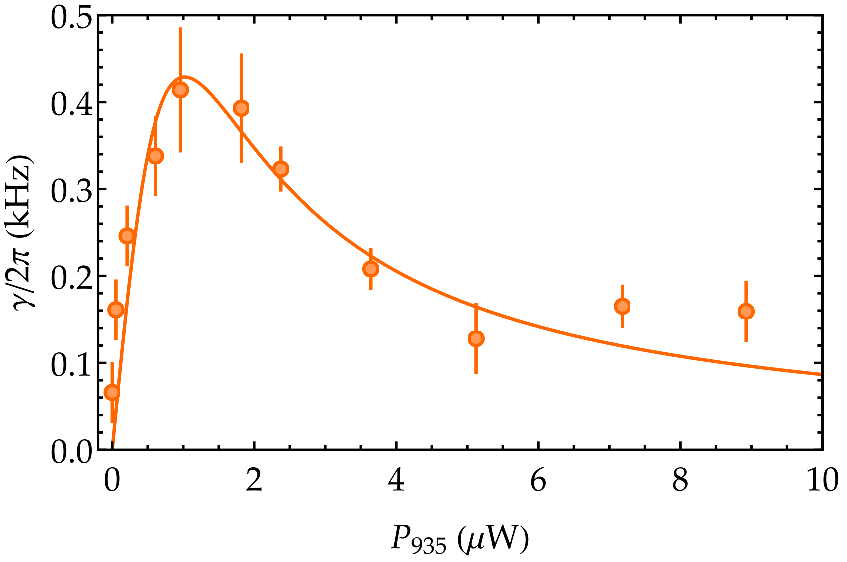

Figure S2: **Cooling rate versus 935 nm power.** An optimal 935 nm power is observed with the measured RSB Rabi frequency of about 3.4 kHz for each 435 nm tone. The solid curve is the theoretical results using the steady state solution of the master equation of a simplified three-level system ($|g\rangle$, $|o\rangle$, and $|e\rangle$) with $\gamma \approx \Gamma_{935}\rho_{ee}$, where Γ_{935} is the scattering rate of $|e\rangle$ and ρ_{ee} its the steady-state population. Here, we use the 935 nm detuning from $|e\rangle$, $\Delta_{935} = 2\pi \times 6.4$ MHz for the theory. A cooling rate baseline of $2\pi \times 0.066$ kHz is observed due to the residual cooling from the 935 nm tone of $^{171}Yb^{+}$ that is constantly on during the experiments.

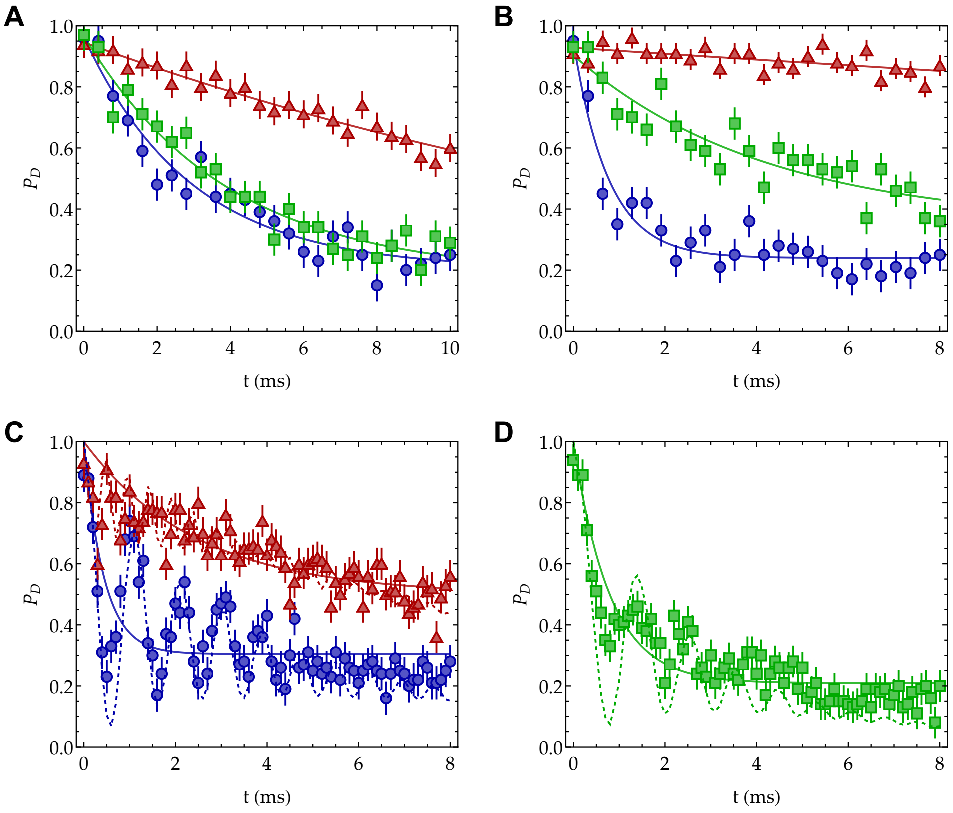

Figure S3: **Donor population dynamics.** (**A** to **D**) Time-evolution traces of the donor population in the (A and B) non-adiabatic (from Figs. 3B and 3E) and (C and D) adiabatic regime (from Fig. 4B). Red triangles, blue circles, and green squares are experimental data for $\Delta E = \omega/2$, $\Delta E = \omega$, and $\Delta E = 2\omega$, respectively. The solid curves are their corresponding exponential fits. The dynamics in the non-adiabatic regime is well described by an exponential decay. Conversely, in the strongly adiabatic regime, the donor population exhibits initial oscillations between the donor and acceptor states, and the exponential fit overestimates both the transfer rate and the final donor population. In (C and D), we also include the numerical results from Eq. (2) in the main text to highlight the oscillatory behavior of the strongly adiabatic regime.

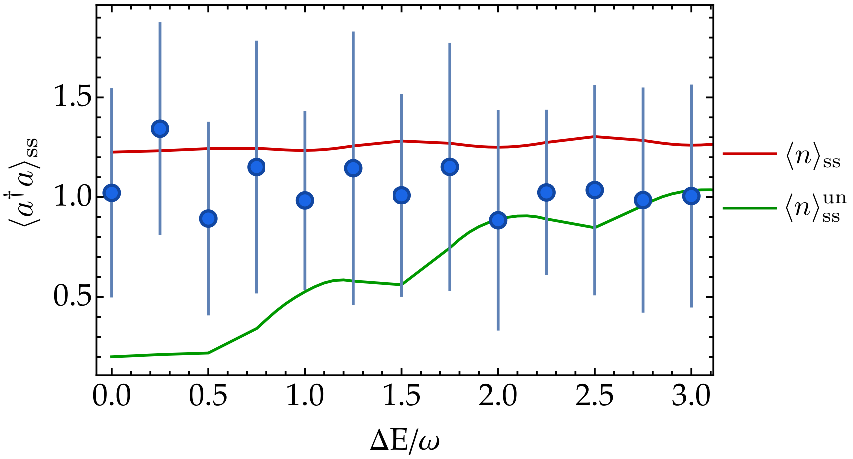

Figure S4: **Characterization of steady-state phonon population.** Average phonon population in the steady state (blue circles) as a function of ΔE using $(V_x, g, \gamma) = (0.19, 1.91, 0.038)\omega$, $\delta/2\pi = -4$ kHz, and $\bar{n} = 0.2$. The average phonon population is extracted by fitting the first six phonon states. The error bars are the standard deviations from the mean. The dark red solid line is the exact prediction given by Eq. (S.13) while the dark green solid line is the prediction given by Eq. (S.16). At low ΔE, there is spin-phonon correlation in the steady state, which decreases monotonically as ΔE is increased. Here, we also consider a motional dephasing of $\gamma_m = 0.0013\omega$.

REFERENCES AND NOTES

1. A. J. Daley, I. Bloch, C. Kokail, S. Flannigan, N. Pearson, M. Troyer, P. Zoller, Practical quantum advantage in quantum simulation. *Nature* **607**, 667–676 (2022).

2. M. Reiher, N. Wiebe, K. M. Svore, D. Wecker, M. Troyer, Elucidating reaction mechanisms on quantum computers. *Proc. Natl. Acad. Sci. U.S.A.* **114**, 7555–7560 (2017).

3. N. Maskara, S. Ostermann, J. Shee, M. Kalinowski, A. M. Gomez, R. A. Bravo, D. S. Wang, A. I. Krylov, N. Y. Yao, M. Head-Gordon, M. D. Lukin, S. F. Yelin, Programmable simulations of molecules and materials with reconfigurable quantum processors. arXiv:2312.02265 [quant-ph] (2023).

4. S. Mostame, J. Huh, C. Kreisbeck, A. J. Kerman, T. Fujita, A. Eisfeld, A. Aspuru-Guzik, Emulation of complex open quantum systems using superconducting qubits. *Quantum Inf. Process.* **16**, 44 (2017).

5. M. Kang, H. Nuomin, S. N. Chowdhury, J. L. Yuly, K. Sun, J. Whitlow, J. Valdiviezo, Z. Zhang, P. Zhang, D. N. Beratan, K. R. Brown, Seeking a quantum advantage with trapped-ion quantum simulations of condensed-phase chemical dynamics. *Nat. Rev. Chem.* **8**, 340–358 (2024).

6. H. Frauenfelder, P. G. Wolynes, Rate theories and puzzles of hemeprotein kinetics. *Science* **229**, 337–345 (1985).

7. R. Zhuravel, H. Huang, G. Polycarpou, S. Polydorides, P. Motamarri, L. Katrivas, D. Rotem, J. Sperling, L. A. Zotti, A. B. Kotlyar, J. C. Cuevas, V. Gavini, S. S. Skourtis, D. Porath, Backbone charge transport in double-stranded DNA. *Nat. Nanotechnol.* **15**, 836–840 (2020).

8. P. G. Wolynes, Imaginary time path integral Monte Carlo route to rate coefficients for nonadiabatic barrier crossing. *J. Chem. Phys.* **87**, 6559–6561 (1987).

9. C. Zheng, J. A. McCammon, P. G. Wolynes, Quantum simulation of nuclear rearrangement in electron transfer reactions. *Proc. Natl. Acad. Sci. U.S.A.* **86**, 6441–6444 (1989).

10. J. E. Lawrence, D. E. Manolopoulos, Path integral methods for reaction rates in complex systems. *Faraday Discuss.* **221**, 9 (2020), 29.

11. E. R. Bittner, P. J. Rossky, Quantum decoherence in mixed quantum-classical systems: Nonadiabatic processes. *J. Chem. Phys.* **103**, 8130–8143 (1995).

12. G. D. Scholes, G. R. Fleming, L. X. Chen, A. Aspuru-Guzik, A. Buchleitner, D. F. Coker, G. S. Engel, R. van Grondelle, A. Ishizaki, D. M. Jonas, J. S. Lundeen, J. K. McCusker, S. Mukamel, J. P. Ogilvie, A. Olaya-Castro, M. A. Ratner, F. C. Spano, K. B. Whaley, X. Zhu, Using coherence to enhance function in chemical and biophysical systems. *Nature* **543**, 647–656 (2017).

13. L. Wang, M. A. Allodi, G. S. Engel, Quantum coherences reveal excited-state dynamics in biophysical systems. *Nat. Rev. Chem.* **3**, 477–490 (2019).

14. Y. Tanimura, Numerically "exact" approach to open quantum dynamics: The hierarchical equations of motion (HEOM). *J. Chem. Phys.* **153**, 020901 (2020).

15. A. Strathearn, P. Kirton, D. Kilda, J. Keeling, B. W. Lovett, Efficient non-Markovian quantum dynamics using time-evolving matrix product operators. *Nat. Commun.* **9**, 3322 (2018).

16. D. Tamascelli, A. Smirne, J. Lim, S. F. Huelga, M. B. Plenio, Efficient simulation of finite-temperature open quantum systems. *Phys. Rev. Lett.* **123**, 090402 (2019).

17. A. D. Somoza, O. Marty, J. Lim, S. F. Huelga, M. B. Plenio, Dissipation-assisted matrix product factorization. *Phys. Rev. Lett.* **123**, 100502 (2019).

18. N. Makri, Modular path integral methodology for real-time quantum dynamics. *J. Chem. Phys.* **149**, 214108 (2018).

19. S. Kundu, N. Makri, Real-time path integral simulation of exciton-vibration dynamics in light-harvesting bacteriochlorophyll aggregates. *J. Phys. Chem. Lett.* **11**, 8783–8789 (2020).

20. D. J. Gorman, B. Hemmerling, E. Megidish, S. A. Moeller, P. Schindler, M. Sarovar, H. Haeffner, Engineering vibrationally assisted energy transfer in a trapped-ion quantum simulator. *Phys. Rev. X* **8**, 011038 (2018).

21. J. Whitlow, Z. Jia, Y. Wang, C. Fang, J. Kim, K. R. Brown, Quantum simulation of conical intersections using trapped ions. *Nat. Chem.* **15**, 1509–1514 (2023).

22. C. H. Valahu, V. C. Olaya-Agudelo, R. J. MacDonell, T. Navickas, A. D. Rao, M. J. Millican, J. B. Pérez-Sánchez, J. Yuen-Zhou, M. J. Biercuk, C. Hempel, T. R. Tan, I. Kassal, Direct observation of geometric-phase interference in dynamics around a conical intersection. *Nat. Chem.* **15**, 1503–1508 (2023).

23. C. S. Wang, N. E. Frattini, B. J. Chapman, S. Puri, S. M. Girvin, M. H. Devoret, R. J. Schoelkopf, Observation of wave-packet branching through an engineered conical intersection. *Phys. Rev. X* **13**, 011008 (2023).

24. A. Potočnik, A. Bargerbos, F. A. Y. N. Schröder, S. A. Khan, M. C. Collodo, S. Gasparinetti, Y. Salathé, C. Creatore, C. Eichler, H. E. Türeci, A. W. Chin, A. Wallraff, Studying light-harvesting models with superconducting circuits. *Nat. Commun.* **9**, 904 (2018).

25. C. Maier, T. Brydges, P. Jurcevic, N. Trautmann, C. Hempel, B. P. Lanyon, P. Hauke, R. Blatt, C. F. Roos, Environment-assisted quantum transport in a 10-qubit network. *Phys. Rev. Lett.* **122**, 050501 (2019).

26. K. Sun, C. Fang, M. Kang, Z. Zhang, P. Zhang, D. N. Beratan, K. R. Brown, J. Kim, Quantum simulation of polarized light-induced electron transfer with a trapped-ion qutrit system. *J. Phys. Chem. Lett.* **14**, 6071–6077 (2023).

27. C. Sparrow, E. Martín-López, N. Maraviglia, A. Neville, C. Harrold, J. Carolan, Y. N. Joglekar, T. Hashimoto, N. Matsuda, J. L. O'Brien, D. P. Tew, A. Laing, Simulating the vibrational quantum dynamics of molecules using photonics. *Nature* **557**, 660–667 (2018).

28. H. Rohde, S. T. Gulde, C. F. Roos, P. A. Barton, D. Leibfried, J. Eschner, F. Schmidt-Kaler, R. Blatt, Sympathetic ground-state cooling and coherent manipulation with two-ion crystals. *J. Opt. B. Quantum Semiclassical Opt.* **3**, S34–S41 (2001).

29. B. B. Blinov, L. Deslauriers, P. Lee, M. J. Madsen, R. Miller, C. Monroe, Sympathetic cooling of trapped Cd^+ isotopes. *Phys. Rev. A* **65**, 040304 (2002).

30. A. J. Leggett, S. Chakravarty, A. T. Dorsey, M. P. A. Fisher, A. Garg, W. Zwerger, Dynamics of the dissipative two-state system. *Rev. Mod. Phys.* **59**, 1 (1987).

31. A. Garg, J. N. Onuchic, V. Ambegaokar, Effect of friction on electron transfer in biomolecules. *J. Chem. Phys.* **83**, 4491–4503 (1985).

32. P. G. Wolynes, Dissipation, tunneling, and adiabaticity criteria for curve crossing problems in the condensed phase. *J. Chem. Phys.* **86**, 1957–1966 (1987).

33. F. Schlawin, M. Gessner, A. Buchleitner, T. Schätz, S. S. Skourtis, Continuously parametrized quantum simulation of molecular electron-transfer reactions. *PRX Quantum* **2**, 010314 (2021).

34. R. J. MacDonell, C. E. Dickerson, C. J. T. Birch, A. Kumar, C. L. Edmunds, M. J. Biercuk, C. Hempel, I. Kassal, Analog quantum simulation of chemical dynamics. *Chem. Sci.* **12**, 9794–9805 (2021).

35. D. Lv, S. An, Z. Liu, J. N. Zhang, J. S. Pedernales, L. Lamata, E. Solano, K. Kim, Quantum simulation of the quantum Rabi model in a trapped ion. *Phys. Rev. X* **8**, 021027 (2018).

36. R. Marcus, N. Sutin, Electron transfers in chemistry and biology. *Biochim. Biophys. Acta* **811**, 265–322 (1985).

37. T. D. Lee, F. E. Low, D. Pines, The motion of slow electrons in a polar crystal. *Phys. Rev.* **90**, 297–302 (1953).

38. G. Clos, D. Porras, U. Warring, T. Schaetz, Time-resolved observation of thermalization in an isolated quantum system. *Phys. Rev. Lett.* **117**, 170401 (2016).

39. G. X. Wang, Y. K. Wu, R. Yao, W. Q. Lian, Z. J. Cheng, Y. L. Xu, C. Zhang, Y. Jiang, Y. Z. Xu, B. X. Qi, P. Y. Hou, Z. C. Zhou, L. He, L. M. Duan, Simulating the spin-boson model with a controllable reservoir in an ion trap. *Phys. Rev. A* **109**, 062402 (2024).

40. A. Lemmer, C. Cormick, D. Tamascelli, T. Schaetz, S. F. Huelga, M. B. Plenio, A trapped-ion simulator for spin-boson models with structured environments. *New J. Phys.* **20**, 073002 (2018).

41. C. Schneider, D. Porras, T. Schaetz, Experimental quantum simulations of many-body physics with trapped ions. *Rep. Prog. Phys.* **75**, 024401 (2012).

42. M. Cetina, L. N. Egan, C. Noel, M. L. Goldman, D. Biswas, A. R. Risinger, D. Zhu, C. Monroe, Control of transverse motion for quantum gates on individually addressed atomic qubits. *PRX Quantum* **3**, 010334 (2022).

43. K. D. Demadis, C. M. Hartshorn, T. J. Meyer, The localized-to-delocalized transition in mixed-valence chemistry. *Chem. Rev.* **101**, 2655–2686 (2001).

44. S. S. Skourtis, A. J. R. Da Silva, W. Bialek, J. N. Onuchic, New look at the primary charge separation in bacterial photosynthesis. *J. Phys. Chem.* **96**, 8034–8041 (1992).

45. J. N. Onuchic, P. G. Wolynes, Classical and quantum pictures of reaction dynamics in condensed matter: Resonances, dephasing, and all that. *J. Phys. Chem.* **92**, 6495–6503 (1988).

46. D. E. Logan, P. G. Wolynes, Dephasing and Anderson localization in topologically disordered systems. *Phys. Rev. B* **36**, 4135–4147 (1987).

47. M. B. Plenio, S. F. Huelga, Dephasing-assisted transport: Quantum networks and biomolecules. *New J. Phys.* **10**, 113019 (2008).

48. P. G. Wolynes, Some quantum weirdness in physiology. *Proc. Natl. Acad. Sci. U.S.A.* **106**, 17247–17248 (2009).

49. P. Rebentrost, M. Mohseni, I. Kassal, S. Lloyd, A. Aspuru-Guzik, Environment-assisted quantum transport. *New J. Phys.* **11**, 033003 (2009).

50. A. W. Chin, A. Datta, F. Caruso, S. F. Huelga, M. B. Plenio, Noise-assisted energy transfer in quantum networks and light-harvesting complexes. *New J. Phys.* **12**, 065002 (2010).

51. D. Tamascelli, A. Smirne, S. F. Huelga, M. B. Plenio, Nonperturbative treatment of non-Markovian dynamics of open quantum systems. *Phys. Rev. Lett.* **120**, 030402 (2018).

52. I. de Vega, D. Alonso, Dynamics of non-Markovian open quantum systems. *Rev. Mod. Phys.* **89**, 015001 (2017).

53. P. M. Harrington, E. J. Mueller, K. W. Murch, Engineered dissipation for quantum information science. *Nat. Rev. Phys.* **4**, 660–671 (2022).

54. S. J. Jang, B. Mennucci, Delocalized excitons in natural light-harvesting complexes. *Rev. Mod. Phys.* **90**, 035003 (2018).

55. A. Ishizaki, G. R. Fleming, Theoretical examination of quantum coherence in a photosynthetic system at physiological temperature. *Proc. Natl. Acad. Sci. U.S.A.* **106**, 17255–17260 (2009).

56. A. Mattioni, F. Caycedo-Soler, S. F. Huelga, M. B. Plenio, Design principles for long-range energy transfer at room temperature. *Phys. Rev. X* **11**, 041003 (2021).

57. C. Monroe, W. C. Campbell, L. M. Duan, Z. X. Gong, A. V. Gorshkov, P. W. Hess, R. Islam, K. Kim, N. M. Linke, G. Pagano, P. Richerme, C. Senko, N. Y. Yao, Programmable quantum simulations of spin systems with trapped ions. *Rev. Mod. Phys.* **93**, 025001 (2021).

58. S. Ding, G. Maslennikov, R. Hablützel, D. Matsukevich, Cross-Kerr nonlinearity for phonon counting. *Phys. Rev. Lett.* **119**, 193602 (2017).

59. C. Zhang, M. Gruebele, D. E. Logan, P. G. Wolynes, Surface crossing and energy flow in many-dimensional quantum systems. *Proc. Natl. Acad. Sci. U.S.A.* **120**, e2221690120 (2023).

60. K. Sun, M. Kang, H. Nuomin, G. Schwartz, D. N. Beratan, K. R. Brown, J. Kim, Quantum simulation of spin-boson models with structured bath. arXiv:2405.14624 [quant-ph] (2024).

61. C. Tamm, N. Huntemann, B. Lipphardt, V. Gerginov, N. Nemitz, M. Kazda, S. Weyers, E. Peik, Cs-based optical frequency measurement using cross-linked optical and microwave oscillators. *Phys. Rev. A* **89**, 023820 (2014).

62. D. T. C. Allcock, W. C. Campbell, J. Chiaverini, I. L. Chuang, E. R. Hudson, I. D. Moore, A. Ransford, C. Roman, J. M. Sage, D. J. Wineland, *omg* blueprint for trapped ion quantum computing with metastable states. *Appl. Phys. Lett.* **119**, 214002 (2021).

63. J. Johansson, P. Nation, F. Nori, QuTiP 2: A Python framework for the dynamics of open quantum systems. *Comput. Phys. Commun.* **184**, 1234–1240 (2013).

64. C. Flühmann, T. L. Nguyen, M. Marinelli, V. Negnevitsky, K. Mehta, J. P. Home, Encoding a qubit in a trapped-ion mechanical oscillator. *Nature* **566**, 513–517 (2019).

65. S. F. Huelga, A. Rivas, M. B. Plenio, Non-Markovianity-assisted steady state entanglement. *Phys. Rev. Lett.* **108**, 160402 (2012).

66. H.-P. Breuer, F. Petruccione, *The Theory of Open Quantum Systems* (Oxford Univ. Press, 2007).

67. H. J. Carmichael, *Statistical Methods in Quantum Optics 1: Master Equations and Fokker-Planck Equations* (Springer Science & Business Media, 2013).

68. T. Brandes, “Quantum dissipation” in *Lectures on Background to Quantum Information* (TU Berlin, 2004).

69. M. Mohseni, Y. Omar, G. S. Engel, M. B. Plenio, *Quantum Effects in Biology* (Cambridge Univ. Press, 2014).

70. M. A. Nielsen, I. L. Chuang, *Quantum Computation and Quantum Information* (Cambridge Univ. Press, 2001), vol. **2**.
